# Ischemic preconditioning enhances 6-hour sprint, jump, and isometric strength responses to complex training in resistance-trained males: a randomized controlled pilot trial

**DOI:** 10.7717/peerj.21649

**Published:** 2026-08-21

**Authors:** Limingfei Zhou, Rou Wang, Yundi Zhang, Lin Wang, Zhenyu Li, Shuang Qin, Guole Jiang

**Affiliations:** 1 School of Strength and Conditioning Training, Beijing Sport University, Beijing, China; 2 Key Laboratory for Performance Training and Recovery of General Administration of Sport, Beijing, China; 3 Sports Coaching College, Beijing Sport University, Beijing, China; 4 School of Economics and Management, University of Science and Technology Beijing, Beijing, China; 5 Sports Teaching and Research Department, China University of Mining and Technology-Beijing, Beijing, China; 6 Longcheng Innovation School, Education Group of Longcheng High School, Guangdong, China; 7 School of Acupuncture-moxibustion, Tuina and Rehabilitation, Hunan University of Chinese Medicine, Changsha, China; 8 Basic Education College, National University of Defense Technology, Changsha, China

**Keywords:** Ischemic preconditioning, Complex training (resistance–plyometric pairing), Delayed performance potentiation (time-course effects), Post-activation performance enhancement (PAPE), Neuromuscular performance, Power performance (jump measures), Sprint speed performance (10 m/30 m), Fatigue recovery/recovery–adaptation timeline, Time-dependent assessment, Randomized controlled pilot trial

## Abstract

**Objective:**

Optimization of the recovery-adaptation timeline following high-intensity training is critical for athletic performance. However, the specific time-course effects of ischemic preconditioning (IPC) when combined with complex training (CT) remain unclear. This study aimed to examine the delayed potentiation effects of IPC applied prior to a CT protocol on power and speed performance over a 48-hour period in resistance-trained males.

**Methods:**

Sixteen resistance-trained males (relative back squat one-repetition maximum (1RM) ≥ 1.5×body mass) with at least one year of resistance training experience participated in this randomized controlled trial. Participants were assigned to either an IPC with CT group (IPC+CT; *n* = 8) or a CT-only control group (CT-only; *n* = 8). The IPC protocol involved five 5-minute cycles of bilateral thigh occlusion (220 mmHg) alternating with 5-minute reperfusion periods, before receiving a lower-body CT intervention. Sprint performance (10-m and 30-m sprint), change-of-direction ability (5-0-5 test), jump performance (standing long jump (SLJ), countermovement jump [CMJ], squat jump (SJ), drop jump (DJ), and continuous vertical jump (CVJ)), maximal strength (1RM back squat), and isometric strength (isometric mid-thigh pull (IMTP)) were assessed at baseline and at 6, 24, and 48 hours post-intervention. This trial was registered at the Chinese Clinical Trial Registry (ChiCTR2500114472).

**Results:**

At 6 h post-intervention, the IPC+CT group showed more favorable responses than the CT-only group in the 10-m sprint, SLJ, CMJ, SJ, DJ, CVJ, and IMTP outcomes (all *P* < 0.045). Bonferroni-adjusted within-group comparisons indicated that the 6 h response was partially attenuated at 24 h and 48 h, although the pattern differed between groups and outcomes. These outcome-specific findings should be interpreted as exploratory because no multiplicity adjustment was applied across the different performance outcomes.

**Conclusions:**

These preliminary findings suggest that IPC applied before CT may promote a short-term improvement in selected power, speed, and isometric strength outcomes, particularly at 6 h post-intervention. Larger sham-controlled studies with physiological measurements are needed to confirm these effects and clarify the underlying mechanisms.

## Introduction

Optimizing neuromuscular performance (*e.g.*, power, maximum strength, and speed) is a core objective of athletic training (Lim & Barley, 2016; Suchomel, Nimphius & Stone, 2016). In the hours and days following high-intensity training, it is crucial to determine whether the exercise fatigue induced by the high training load can lead to supercompensation and enhanced athletic performance without increasing the risk of injury (Gabbett, 2016; Cunanan et al., 2018). Therefore, effective strategies are needed to accelerate recovery and support subsequent improvements in power and speed performance (Dupuy et al., 2018).

Complex training (CT) (*i.e.,* combines high-intensity resistance training with biomechanically similar plyometric training) is a common training method for improving power and speed (Freitas et al., 2017; Junge, Jørgensen & Nybo, 2023), and can induce the post-activation performance enhancement (PAPE) phenomenon to improve acute athletic performance in power and speed at different time points (6–48 h) after training (Blazevich & Babault, 2019; Harrison et al., 2019). The effect of CT is determined by the balance between fatigue and potentiation. We posit that IPC may shift this balance by accelerating fatigue recovery, thereby allowing the potentiation effects to manifest earlier (*e.g.*, at 6 h) than under normal recovery conditions (Docherty & Sporer, 2000). Therefore, efforts have been put to develop effective method during CT intervention that can accelerate fatigue recovery efficiency without affecting the improvement of athletic performance.

Ischemic preconditioning (IPC) is one such promising strategy involving brief cycles of blood flow restriction and reperfusion to improve athletic performance and promote recovery (Incognito, Burr & Millar, 2016; Salvador et al., 2016; Mănescu et al., 2025). Although blood biomarkers were not measured in this study, previous research suggests that IPC may trigger the release of nitric oxide (NO), which regulates mitochondrial oxygen consumption and reduces the production of reactive oxygen species (ROS), thereby mitigating oxidative damage to muscle cells (Rassaf et al., 2014; Paradis-Deschênes, Joanisse & Billaut, 2017; Ceylan, Taşkın & Šimenko, 2023). This mechanism may upregulate the expression of inducible nitric oxide synthase, leading to increased NO bioavailability and reduced ROS levels in mitochondria, both of which contribute to improved athletic performance (Salvador et al., 2016; Sprick & Rickards, 2017; Chen et al., 2025; Wu et al., 2025a). Recent studies have increasingly examined IPC as a potential strategy for improving exercise performance and recovery (Patterson et al., 2019). For example, IPC has been reported to activate endogenous protective mechanisms in two distinct phases: an early phase, beginning immediately after IPC and lasting approximately 6 h, and a late phase, beginning approximately 24 h after IPC and lasting up to 72 h (Cruz et al., 2015; Fan, Fu & Wu, 2025; Wu et al., 2025a; Wu et al., 2025b). These time points (6 h, 24 h, and 48 h) were selected to align with the physiological timelines of phosphocreatine resynthesis, the typical onset of delayed onset muscle soreness (DOMS), and the windows of IPC-induced early (immediate to ∼6 h) and late (∼24 h to 72 h) phases of protection (Cheung, Hume & Maxwell, 2003; De Groot et al., 2010; Niu et al., 2024).

Although the mechanisms and applications of CT and IPC have been examined independently, their potential combined effects remain largely unexplored. First, most previous studies have primarily focused on acute or short-term performance responses, whereas the delayed recovery-performance time course over the subsequent 24–48 h remains insufficiently characterized (Yang et al., 2026). Second, although IPC and CT have been examined separately, their combined effects on delayed neuromuscular performance have rarely been investigated (Page, Swan & Patterson, 2017).

This gap is important from both practical and physiological perspectives. In applied sport settings, coaches are often concerned not only with immediate performance enhancement, but also with whether an intervention can improve readiness for the next training session or competition within the following 24–48 h, especially during high-frequency training periods or multi-day competitions (Halson, 2014; Wehbe et al., 2015). In addition, IPC has been reported to induce time-dependent protective responses, including an early phase and a later phase that may emerge approximately 24 h after the intervention and persist for up to 72 h (Seeger et al., 2017; Lang & Kim, 2022). Moreover, recent evidence suggests that IPC may influence post-exercise recovery and subsequent performance (Arriel et al., 2018), but evidence regarding the delayed effects of IPC when combined with CT remained insufficient (Lopes et al., 2018). Accordingly, further research is needed to compare IPC combined with CT *versus* CT alone across multiple post-intervention time points. Such evidence may help clarify whether IPC can modify the recovery–potentiation timeline following CT and enhance delayed neuromuscular performance.

Therefore, the purpose of this study was to examine whether IPC applied prior to CT influences the delayed time course of lower-limb power and speed performance over a 48-hour period. We hypothesized that ischemic preconditioning performed prior to complex training would enhance lower-limb neuromuscular performance, particularly sprint and jump performance, over the 24–48-hour recovery period compared with complex training alone.

## Materials & Methods

### Participants and study design

The study population comprised sixteen healthy males with a minimum of two years of consistent strength training experience. The inclusion criteria were as follows: (1) no history of cardiovascular, metabolic, or neuromuscular diseases; (2) free from any musculoskeletal injuries in the six months preceding the study; (3) possessing at least one year of experience in complex training and a back squat one-repetition maximum (1RM) of at least 1.5 times their body weight. Exclusion criteria included the use of any medication or dietary supplements known to affect heart rate, blood pressure, or physical performance within 24 h of testing. All participants provided written informed consent after being fully informed of the experimental procedures and potential risk. Furthermore, prior use of IPC was an explicit exclusion criterion. Participants were required to avoid strenuous physical activity for at least 48 h before the intervention and throughout the 48-h follow-up period, while maintaining their habitual sleep routine (with a minimum target of ≥7 h per night) and usual dietary patterns across all testing sessions. This investigation received ethical approval from the Sports Science Experiment Ethics Committee of Beijing Sport University (Approval No. 2024433H). This trial was registered at the Chinese Clinical Trial Registry (ChiCTR2500114472). The experimental procedures adhered strictly to the principles outlined in the Declaration of Helsinki.

**Figure 1:**
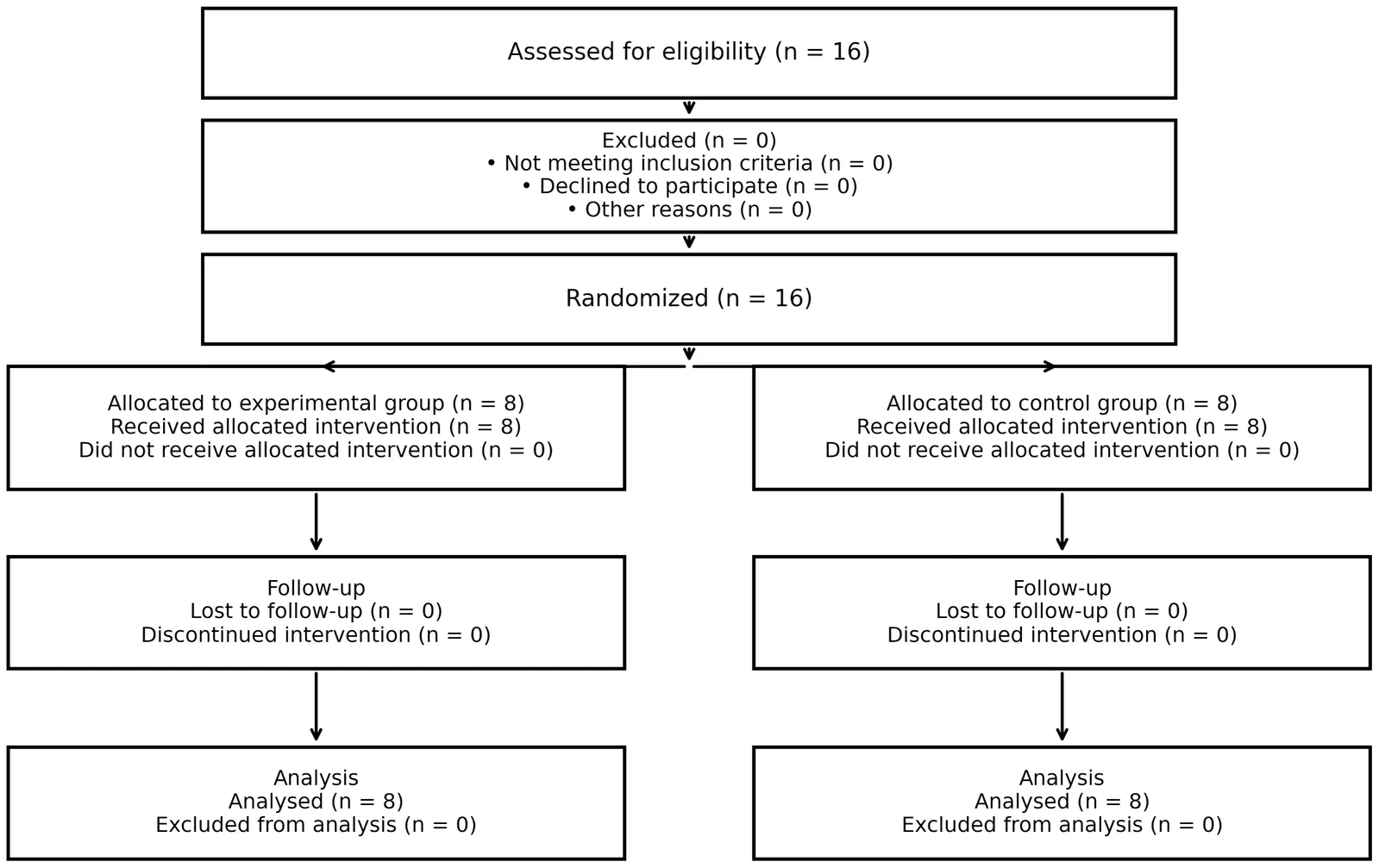
CONSORT flow diagram.

An a priori sample size calculation was performed using G*Power software (version 3.1.9.7) prior to participant recruitment. The analysis was based on an F-test for repeated-measures analysis of variance (ANOVA) assessing the within–between interaction, with a significance level of α = 0.05, statistical power of 1–β = 0.80, and a target effect size of *f* = 0.30, which was selected based on prior related work reporting a moderate effect. The calculation indicated that 14 participants would be required. Therefore, 16 participants were recruited to allow for potential attrition while maintaining adequate statistical power for this pilot trial (Beck, 2013). Participant flow is shown in Fig. 1. Demographic and baseline performance characteristics of the cohort are summarized in Table 1. An initial analysis confirmed cohort homogeneity, with no statistically significant differences observed in age, body mass, height, weekly physical activity duration, or absolute and relative 1RM back squat performance.

**Figure 2:**
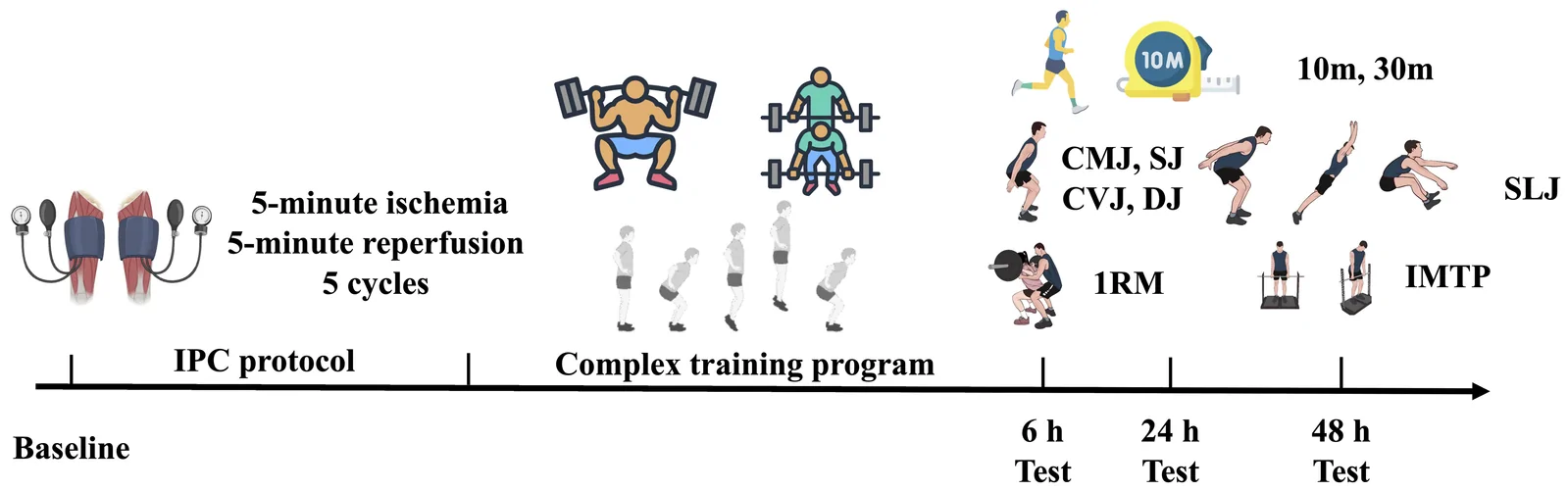
The training protocol and assessment of IPC and CT intervention program.

A randomized controlled trial was conducted to investigate whether IPC applied immediately before a complex training (CT) session could augment the delayed effects on neuromuscular performance (Table 2). Participants were randomly assigned to either an experimental group (IPC+CT) or a control group (CT-only). Group allocation was generated using a computer-based random-number sequence by a researcher not involved in outcome testing or intervention delivery. Allocation concealment was maintained using sealed, opaque envelopes, which were opened only after completion of baseline assessments. This assessor was blinded to group allocation throughout data collection and outcome assessment. Participants were instructed not to disclose their group assignment to the assessor. While a sham-IPC group was not included, the CT-only group serves as an active control representing standard training practice, allowing for the evaluation of the additive benefits of IPC in a practical setting. Performance was assessed at baseline and subsequently at 6, 24, and 48 h post-intervention using a comprehensive test battery. Both groups performed the identical CT protocol, which is detailed in Table 2. The IPC+CT group underwent a standardized IPC protocol immediately prior to commencing the CT session. The IPC protocol was administered with participants in a supine position. Non-elastic pneumatic cuffs (width: 14 cm; Theratools, China) were applied bilaterally to the most proximal aspect of the thighs, just distal to the gluteal fold (Patterson et al., 2021). The intervention consisted of five cycles of 5 min of ischemia, induced by rapid cuff inflation to 220 mmHg, each followed by a 5-minute reperfusion period where the cuffs were fully deflated. The entire procedure lasted 50 min. The full IPC procedure is illustrated in Fig. 2. The selection of 220 mmHg was based on previous IPC studies using standardized fixed cuff pressures to ensure complete arterial occlusion in the lower limbs and to elicit systemic physiological responses under comparable experimental conditions (Bailey et al., 2012; Sabino-Carvalho et al., 2017). This pressure was applied as a standardized protocol for all participants and was not individualized according to arterial occlusion pressure (AOP).

**Table 1:** Descriptive statistics of participants.

	Height (cm)	Body mass (kg)	Age (y)
CT-only	178.35 ± 6.27	75.21 ± 5.48	19.35 ± 1.60
IPC+CT	178.70 ± 6.75	76.09 ± 5.75	19.00 ± 1.27

**Table 2:** The main part of complex training intervention program.

**Intervention**	**Intensity**	**Sets × repetitions**	**Interval**
Squat + squat jump	80% 1RM + body weight	3 ×(4–6 + 10–12)	4 min
Deadlift + power shrug from floor	80% 1RM + 40% 1RM	3 ×(4–6 + 10–12)	4 min
Weight-bearing lunge + split-leg squat jump	80% 1RM + body weight	3 ×(4–6 + 10–12)	4 min

### Measurements

Assessed variables comprised: 10 m, 30 m, 5-0-5, standing long jump (SLJ), squat jump (SJ), countermovement jumps (CMJ), drop jump (DJ), and continuous vertical jump (CVJ). Maximal strength was evaluated *via* one-repetition maximum (1RM) squat and isometric mid-thigh pull (IMTP). Each participant performed all measurements at every assessment point (baseline, 6 h, 24 h, and 48 h post-intervention). A recovery period of 5 to 10 min was provided between trials. The CT-only group was selected as an active control to reflect standard training practice. Although the absence of a sham-IPC condition limits the ability to control for placebo effects, the CT-only comparison allowed us to evaluate whether adding IPC provided additional benefits beyond CT alone. A detailed schematic of the experimental timeline and procedures for both the IPC+CT and CT-only interventions is provided in Fig. 2. These pre-test restrictions were maintained before each testing session. Participants were asked to have eaten some food within the 4 h preceding the testing. Before assessment, participants performed a 5-minute warm-up (low-intensity running, high skipping, leg flexions, lateral running, front and behind arm rotation, and sprints). Afterward, participants completed 5 min of stretching exercises and were familiarized with each assessment by performing 1–3 practice trials.

### Speed test

A straight 30-meter course was meticulously measured and marked. Three pairs of dual-beam photocell gates (Smartspeed, Fusion Sport, Brisbane, Australia) were positioned at the start line (0 m), the 10 m mark, and the 30 m mark. The gates were set at a height of 1.0 meter to ensure consistent triggering by the participant’s body. Participants initiated each sprint from a stationary, split-stance position, with their front foot placed 0.5 m behind the starting line to prevent any rocking motion from prematurely triggering the first gate. Upon a verbal command (“Ready... Go!”), participants were instructed to sprint maximally through the entire 30-meter distance, ensuring they did not decelerate before passing the final timing gate. The electronic timing system automatically recorded the split time at 10 m and the final time at 30 m to the nearest 0.01 s. Each participant performed three maximal sprint trials. A minimum of 3 min of passive recovery was mandated between each trial to allow for near-complete restoration of the phosphagen system. The fastest time for both the 10 m and 30 m distances from the three trials was recorded and utilized for subsequent statistical analysis. Encouragement was provided consistently across all trials and for all participants to promote maximal effort.

Change-of-direction (COD) ability was assessed using the 5-0-5 agility test. The protocol required participants to accelerate maximally over a 10-meter run-in before entering a 5-meter timed zone. Upon entering the timed zone, they sprinted 5 m to a designated line, executed a 180° turn, and sprinted back 5 m through the timing gates. The test was performed separately for both left and right turning directions. To ensure consistency, participants were instructed to plant with a specified foot at the COD line for all trials of a given direction. The starting position was standardized, with participants adopting a staggered stance 0.5 m behind the initial timing gate, aligned with their hip. A dual-beam electronic timing system (Smartspeed, Fusion Sport, Brisbane, Australia) was used to record the time to the nearest 0.01 s. Two trials were completed for each turning direction, interspersed with a 3-minute passive recovery period. The fastest time for each turning direction was retained, and the mean of the left- and right-turning times was used for subsequent analysis.

### Jump test

Explosive lower-body power was assessed using the SLJ test. The test was conducted on a non-slip, hard surface where a take-off line was clearly marked. A certified steel tape measure was affixed to the floor, perpendicular to the take-off line, to ensure measurement accuracy. Following standardized instructions, participants stood with their feet shoulder-width apart and toes behind the take-off line. They were permitted to perform a preparatory countermovement by swinging their arms backward while simultaneously flexing at the hips, knees, and ankles, before generating a maximal horizontal jump. Each participant performed three maximal trials, interspersed with 1–2 min of passive recovery. Jump distance was measured to the nearest one cm from the take-off line to the rearmost point of contact upon landing (typically the heels). In the event of a backward fall, the point of contact closest to the take-off line (*e.g.*, a hand) was used for measurement. The longest distance recorded from the three trials was retained for analysis.

The vertical jump tests consisted of CMJ, SJ, DJ, and CVJ following previous protocols. To perform the CMJ, participants began from an upright position with hands on their hips, rapidly executing a downward movement of self-determined depth followed by a maximum vertical jump, keeping their legs straight throughout the flight. For the SJ, participants were instructed to hold a static squat position with 90° knee flexion for 3 s before jumping. For the DJ, participants stood on a raised platform (*e.g.*, a 30-cm box), stepped off (not jumped), and upon landing, immediately performed an explosive vertical jump, aiming for maximal jump height with minimal ground contact time. The hands were required to be kept on the hips throughout the entire DJ test. The CVJ required participants to perform five consecutive maximal vertical jumps, minimizing ground contact time between each jump while attempting to achieve maximum height on every rebound. Two submaximal practice trials with 1 min of recovery were performed before each jump test type. For all test conditions, three formal trials with 2 min of recovery were completed, and the best performance was used for subsequent analysis. The jump height was calculated from the flight time data derived from a jump mat (Smart Jump, Fusion Sport, Brisbane, Australia).

### 1RM strength test

Maximal lower-body strength was assessed *via* a one-repetition maximum (1RM) back squat test, performed according to established protocols within a power rack (Hammer Strength, Rosemont, IL, USA). Following a general warm-up, participants completed a specific, progressive warm-up protocol. This consisted of sets of 10 repetitions at approximately 50%, five at 70%, three at 80%, and a single repetition at 90% of their estimated 1RM. The initial 1RM was estimated by the research staff based on the participant’s training history, body mass, and age. Subsequently, participants performed three to five maximal attempts to determine their true 1RM, with 3 to 5 min of passive recovery between each attempt. The load was progressively increased after each successful lift until the participant was unable to complete a full repetition. A successful lift was defined as the participant squatting to a depth where the femur was parallel to the floor, as visually verified by the research team. For safety, two trained spotters monitored the participant throughout all maximal attempts. The heaviest successfully lifted load was recorded as the participant’s 1RM.

### IMTP test

Isometric strength characteristics were evaluated using the isometric mid-thigh pull (IMTP) test. Prior to testing, the barbell height was individualized for each participant. The bar was positioned at the midpoint between the greater trochanter and the lateral epicondyle of the femur and secured in a power rack. Participants were instructed to adopt a self-selected, comfortable deadlift stance. The use of an overhand, mixed, or hook grip was permitted. To execute the test, participants were instructed to “pull as hard and as fast as possible” for a 6-second duration upon a verbal command (“3, 2, 1, GO!”). To prevent pre-loading, they were explicitly instructed to remain relaxed and avoid generating any force on the bar prior to the command. Two to three maximal trials were performed, with 2–3 min of passive recovery between each attempt. Force-time data were collected at a sampling rate of 1000 Hz using a uni-axial force plate (Kistler 9281CA, Kistler, Winterthur, Switzerland). The trial with the highest peak force was selected for analysis.

### Statistical analysis

Data were analyzed using JASP software (version 0.18.3, JASP Team, The Netherlands). Descriptive statistics are presented as mean ± standard deviation. Normality was assessed using the Shapiro–Wilk test. Sphericity was examined using Mauchly’s test, and the Greenhouse–Geisser correction was applied when the assumption of sphericity was violated. A two-way repeated-measures ANOVA was used to examine the effects of group (IPC+CT *vs.* CT-only), time (pre-intervention, 6 h, 24 h, and 48 h post-intervention), and their interaction. Partial eta-squared (*η*p^2^) was calculated as the effect-size index for ANOVA results.

When significant interaction effects were detected, post hoc simple-effect analyses were conducted. Within-group pairwise comparisons across the four time points were performed using two-sided paired t tests. Standardized effect sizes for these paired comparisons were calculated as Cohen’s dz, defined as the mean paired difference divided by the standard deviation of the paired differences. The corresponding 95% confidence intervals were obtained by inversion of the noncentral t distribution, thereby accounting for the paired structure of the repeated observations. Bonferroni correction was applied across the six possible pairwise time comparisons within each group and outcome. The 95% confidence intervals for Cohen’s dz were not multiplicity-adjusted and are presented as measures of estimation uncertainty. Because no adjustment was applied across the different performance outcomes, outcome-specific findings were considered exploratory. Statistical significance was set at *P* < 0.05 after the specified Bonferroni correction.

## Results

**Figure 3:**
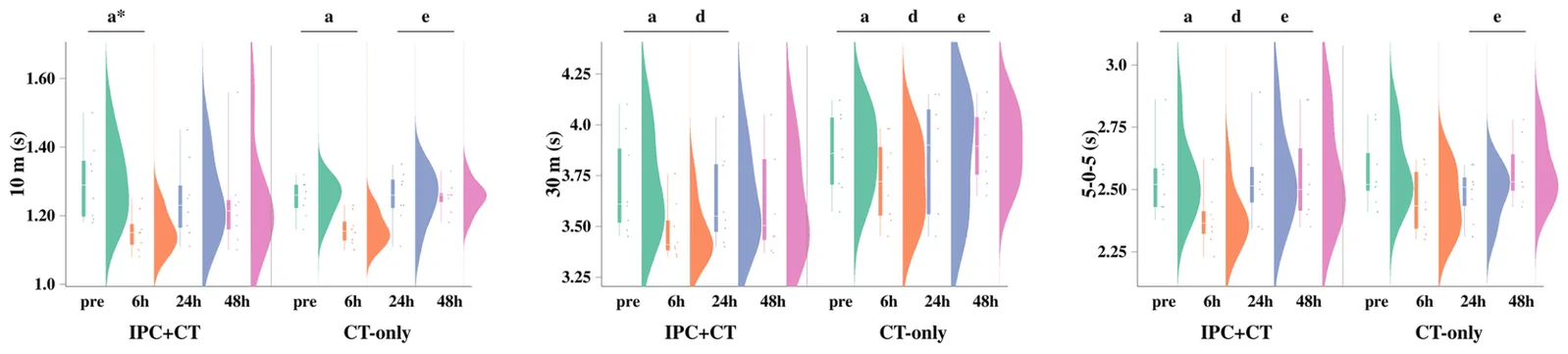
Changes in speed and change of direction performance. *-Statistically significant difference between IPC+CT group and CT-only group, *P* < 0.05; (A) statistically significant difference between pre- and 6 h-test, *P* < 0.05; (B) statistically significant difference between pre- and 24 h-test, *P* < 0.05; (C) statistically significant difference between pre- and 48 h-test, *P* < 0.05; (D) statistically significant difference between 6 h- and 24 h-test, *P* < 0.05; (E) statistically significant difference between 6- and 48 h-test, *P* < 0.05; (F) statistically significant difference between 24 h- and 48 h-test, *P* < 0.05.

**Figure 4:**
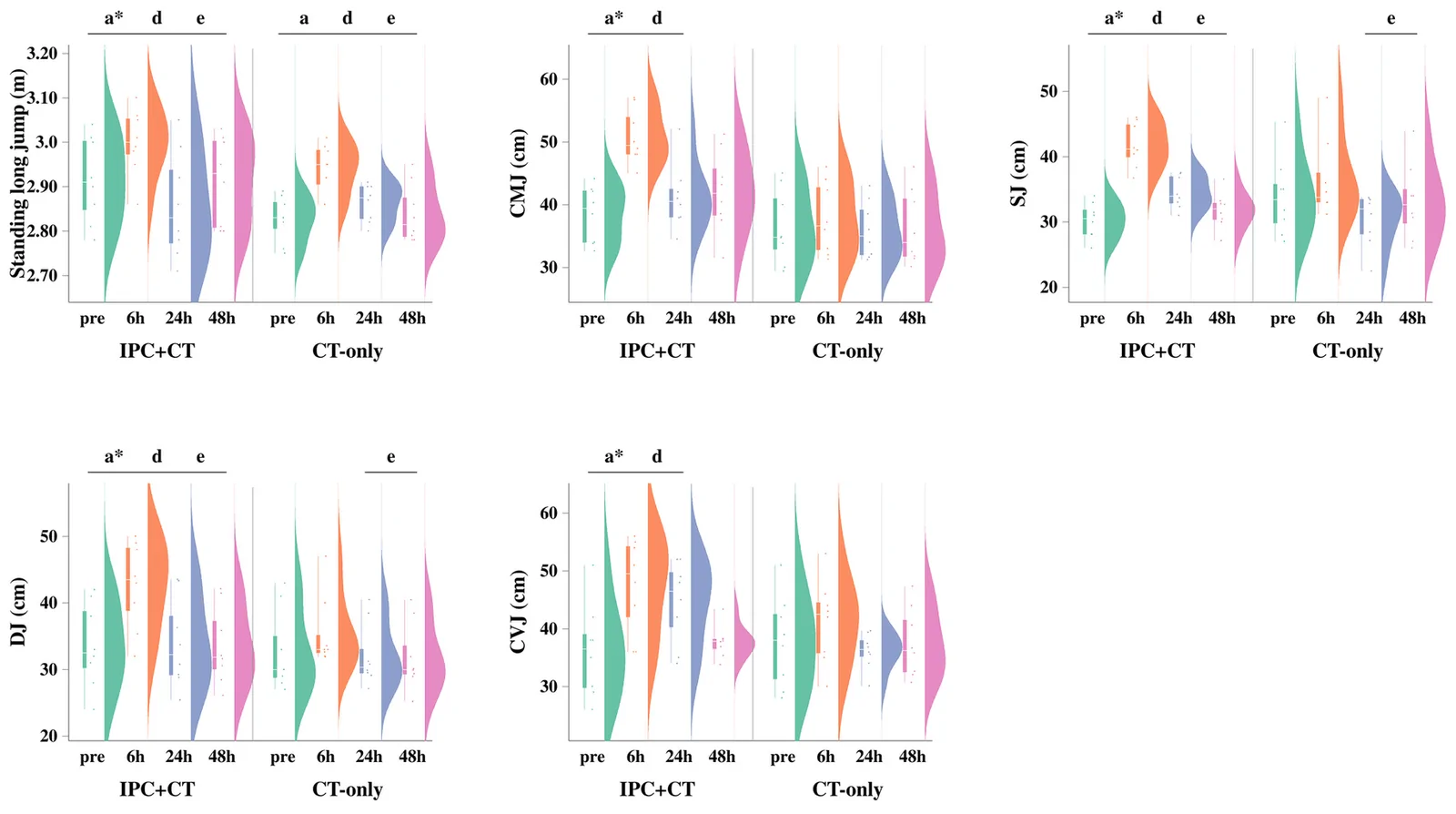
Changes in power performance. *-Statistically significant difference between IPC+CT group and CT-only group, *P* < 0.05; a-Statistically significant difference between pre- and 6 h-test, *P* < 0.05; (B) statistically significant difference between pre- and 24 h-test, *P* < 0.05; (C) statistically significant difference between pre- and 48 h-test, *P* < 0.05; (D) statistically significant difference between 6 h- and 24 h-test, *P* < 0.05; (E) statistically significant difference between 6- and 48 h-test, *P* < 0.05; (F) statistically significant difference between 24h- and 48h-test, *P* < 0.05.

**Figure 5:**
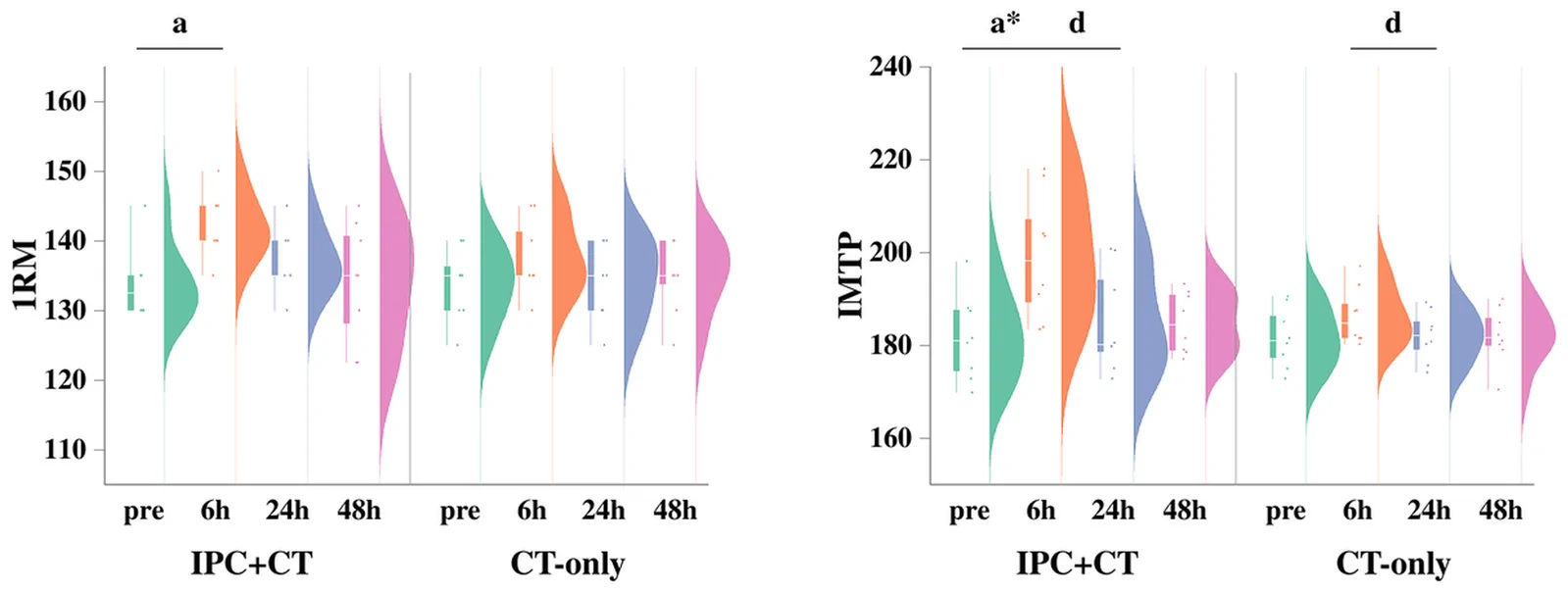
Changes in strength performance. *-Statistically significant difference between IPC+CT group and CT-only group , *P* < 0.05; (A) statistically significant difference between pre- and 6 h-test, *P* < 0.05; (B) statistically significant difference between pre- and 24 h-test, *P* < 0.05; (C) statistically significant difference between pre- and 48 h-test, *P* < 0.05; (D) statistically significant difference between 6 h- and 24 h-test, *P* < 0.05; (E) statistically significant difference between 6- and 48 h-test, *P* < 0.05; (F) statistically significant difference between 24 h- and 48 h-test, *P* < 0.05.

All 16 participants completed the study, and all observations were included in the analysis. The groups did not differ significantly in demographic characteristics or baseline outcome measures (all *P* > 0.695). Performance changes in the IPC+CT and CT-only groups are shown in Figs. 3–5. Detailed pairwise results, including Bonferroni-adjusted *P* values, are presented in Table 3. Bonferroni-adjusted pre-to-6 h comparisons are shown in Table 3. Significant pre-to-6 h changes were observed in the IPC+CT group for the 10-m sprint, 30-m sprint, 5-0-5 test, SLJ, CMJ, SJ, DJ, CVJ, 1RM back squat, and IMTP, and in the CT-only group for the 10-m sprint, 30-m sprint, and SLJ.

**Table 3:** The outcome assessment results for IPC+CT group and CT-only group before and after intervention.

	IPC+CT group				CT-only group			
	Pre	6 h	24 h	48 h	Pre	6 h	24 h	48 h
10-m sprint (s)	1.30 ± 0.11	1.15 ± 0.06 ^a^^*^	1.25 ± 0.12	1.24 ± 0.14	1.25 ± 0.05	1.16 ± 0.05 ^a^	1.25 ± 0.08	1.25 ± 0.05 ^c^
30-m sprint (s)	3.70 ± 0.24	3.47 ± 0.14 ^a^	3.63 ± 0.23 ^b^	3.62 ± 0.26	3.86 ± 0.20	3.73 ± 0.21 ^a^	3.84 ± 0.28 ^b^	3.90 ± 0.18 ^c^
5-0-5 (s)	2.54 ± 0.15	2.38 ± 0.12 ^a^	2.54 ± 0.18 ^b^	2.56 ± 0.20 ^c^	2.57 ± 0.15	2.45 ± 0.13	2.49 ± 0.11	2.57 ± 0.13 ^c^
SLJ (m)	2.92 ± 0.10	3.00 ± 0.07 ^a^^*^	2.86 ± 0.12 ^b^	2.91 ± 0.10 ^c^	2.83 ± 0.05	2.94 ± 0.06 ^a^	2.86 ± 0.04 ^b^	2.84 ± 0.07 ^c^
CMJ (cm)	38.31 ± 1.74	45.01 ± 4.04 ^a^^*^	40.31 ± 5.67 ^b^	39.55 ± 3.96	37.21 ± 2.49	40.03 ± 4.70	39.87 ± 6.09	37.62 ± 4.91
SJ (cm)	33.81 ± 5.96	41.76 ± 3.41 ^a^^*^	34.50 ± 2.47 ^b^	31.77 ± 2.76 ^c^	30.21 ± 2.70	36.48 ± 6.04	30.36 ± 4.04	33.11 ± 5.84 ^c^
DJ (cm)	33.63 ± 6.30	42.67 ± 6.53 ^a^^*^	33.89 ± 6.72 ^b^	33.54 ± 5.83 ^c^	32.62 ± 6.07	35.39 ± 5.37	32.08 ± 4.75	31.80 ± 5.12 ^c^
CVJ (cm)	36.13 ± 8.06	47.50 ± 8.11 ^a^^*^	38.63 ± 7.09 ^b^	37.64 ± 2.78	37.75 ± 7.92	41.13 ± 7.22	36.12 ± 3.08	37.42 ± 6.01
1RM of Squat (kg)	133.75 ± 7.44	141.88 ± 4.58 ^a^	136.88 ± 4.58	134.06 ± 8.55	135.75 ± 5.18	137.50 ± 5.35	134.38 ± 7.63	135.00 ± 4.45
IMTP (kg)	184.11 ± 10.23	199.14 ± 13.55 ^a^^*^	185.10 ± 11.08 ^b^	184.81 ± 6.54	181.61 ± 9.31	186.27 ± 6.12	181.93 ± 5.40 ^b^	181.77 ± 3.14

**Notes.**

All significance markers indicate Bonferroni-adjusted *P* < 0.05.

* IPC+CT group *vs* CT-only group.

a pre-test *vs* 6 h test.

b 6 h *vs* 24 h tests.

c 6 h *vs* 48 h tests.

Significant group × time interactions were observed for the 10-m sprint, SLJ, CMJ, SJ, DJ, CVJ, and IMTP outcomes (all *P* ≤ 0.045; partial eta-squared = 0.172–0.393). At 6 h post-intervention, the IPC+CT group showed more favorable responses than the CT-only group in these outcomes. Because multiple performance outcomes were examined, these outcome-specific findings should be interpreted as exploratory. Bonferroni-adjusted follow-up comparisons indicated that the 6 h response in the IPC+CT group was partially attenuated at 24 h and 48 h across several sprint, change-of-direction, jump, and strength outcomes. In the CT-only group, selected sprint and jump outcomes also differed between 6 h and the later time points, but the response pattern was less consistent.

Bonferroni-adjusted follow-up within-group comparisons showed that, within the IPC+CT group, performance at 6 h differed significantly from 24 h for the 30-m sprint (adjusted *P* = 0.015, dz = −1.623, 95% confidence interval (CI) [−2.685 to −0.519]), 5-0-5 test (*P* = 0.012, dz = −1.705, 95% CI [−2.799 to −0.568]), SLJ (adjusted *P* = 0.004, dz = 2.005, 95% CI [0.747–3.225]), CMJ (adjusted *P* = 0.036, dz = 1.372, 95% CI [0.362–2.337]), SJ (adjusted *P* < 0.001, dz = 6.891, 95% CI [3.307–10.483]), DJ (adjusted *P* = 0.006, dz = 1.926, 95% CI [0.700–3.112]), CVJ (adjusted *P* = 0.002, dz = 2.307, 95% CI [0.920–3.658]), and IMTP (adjusted *P* = 0.006, dz = 1.910, 95% CI [0.691–3.089]). Significant differences were also observed between 6 h and 48 h for the 5-0-5 test (*P* = 0.014, dz = −1.637, 95% CI [−2.703 to −0.527]), SLJ (*P* = 0.017, dz = 1.590, 95% CI [0.498–2.639]), SJ (*P* < 0.001, dz = 2.980, 95% CI [1.292–4.641]), and DJ (*P* = 0.001, dz = 2.509, 95% CI [1.033–3.951]) (Fig. 3).

Within the CT-only group, Bonferroni-adjusted follow-up comparisons showed significant differences between 6 h and 24 h for the 30-m sprint (*P* = 0.046, dz = −1.309, 95% CI [−2.252 to −0.322]), SLJ (*P* = 0.046, dz = 1.308, 95% CI [0.321–2.250]), and IMTP (*P* = 0.008, dz = 1.807, 95% CI [0.630–2.943]). Significant differences were also observed between 6 h and 48 h for the 30-m sprint (*P* < 0.001, dz = −4.265, 95% CI [−6.545 to −1.971]), 10-m sprint (*P* = 0.009, dz = −1.800, 95% CI [−2.933 to −0.625]), 5-0-5 test (*P* = 0.012, dz = −1.702, 95% CI [−2.795 to −0.566]), SLJ (*P* = 0.013, dz = 1.678, 95% CI [0.552–2.761]), SJ (*P* = 0.024, dz = 1.487, 95% CI [0.434–2.496]), and DJ (*P* = 0.037, dz = 1.370, 95% CI [0.360–2.334]) (Figs. 4, 5).

## Discussion

The main finding of this randomized controlled pilot trial was that IPC applied before CT was associated with more favorable neuromuscular performance at 6 h post-intervention, particularly in selected sprint, jump, and isometric strength outcomes. These results suggest that IPC may modify the short-term recovery–potentiation balance following CT, allowing performance benefits to emerge earlier than with CT alone. However, given the pilot design and small sample size, these findings should be interpreted as preliminary.

At 6 h post-intervention, the IPC+CT group showed more favorable responses in selected sprint, jump, and isometric strength outcomes. One plausible interpretation is that IPC may have modified the balance between fatigue and post-activation performance enhancement during early recovery (Blazevich & Babault, 2019; Harrison et al., 2019; Docherty & Sporer, 2000). Previous studies have proposed that IPC may influence peripheral perfusion, muscle oxygenation, and post-exercise recovery (Paradis-Deschênes, Joanisse & Billaut, 2017; Ceylan, Taşkın & Šimenko, 2023; Page, Swan & Patterson, 2017; Patterson et al., 2021); however, these variables were not directly measured in the present study. Therefore, the current performance data should not be interpreted as direct evidence of these physiological mechanisms.

The more favorable responses in CMJ, SJ, DJ, CVJ, and IMTP may also be consistent with better preservation of neuromuscular readiness during the early recovery period. The less pronounced 1RM back squat response at 6 h suggests that rapid force-production tasks and maximal dynamic strength may not follow the same recovery profile after CT (Suchomel, Nimphius & Stone, 2016; Harrison et al., 2019; Dupuy et al., 2018). This pattern could reflect differences in task complexity, stretch-shortening-cycle contribution, neural drive, or peripheral mechanical fatigue, but the absence of direct neural, metabolic, hemodynamic, muscle-oxygenation, or muscle-damage measurements prevents differentiation among these explanations. Accordingly, the mechanistic interpretations in this study should be regarded as plausible explanations rather than confirmed mechanisms. From an applied perspective, IPC may be useful when athletes are required to regain neuromuscular readiness within a short recovery window following high-intensity training (Halson, 2014; Wehbe et al., 2015). Future sham-controlled studies incorporating physiological measurements are needed to determine whether IPC can reliably alter the recovery–potentiation timeline following complex training.

However, the 1RM back squat response at 6 h was less pronounced than the responses observed for several power outcomes. This may reflect differences in task demands and recovery characteristics between explosive and maximal-strength tasks (Suchomel, Nimphius & Stone, 2016; Harrison et al., 2019). Because neural drive, metabolic fatigue, and muscle damage were not measured, the mechanisms underlying the IMTP and 1RM findings cannot be determined. Accordingly, these findings should be interpreted as performance outcomes rather than evidence of preserved central drive. Accordingly, practitioners may consider IPC prior to CT when the goal is not merely immediate performance enhancement, but improved readiness for a subsequent session within the same day or within the next 24–48 h.

At 6 h post-intervention, the IPC+CT group showed a more favorable response in the 10-m sprint than the CT-only group. One possible explanation is that IPC altered the balance between fatigue and performance enhancement during early recovery. Previous studies suggest that IPC may influence peripheral perfusion, muscle oxygenation, and the recovery of high-energy phosphate availability; however, these variables were not measured in the present study. The current findings therefore cannot establish whether these pathways contributed to the observed sprint response. The more favorable CMJ and DJ responses at 6 h may also be consistent with better preservation of stretch-shortening-cycle performance during early recovery. High-intensity CT can produce transient fatigue and muscle damage, whereas IPC has been proposed to attenuate some aspects of post-exercise fatigue. Nevertheless, the present performance data do not directly demonstrate changes in muscle damage, neuromuscular function, or excitation-contraction coupling. These mechanisms should therefore be regarded as plausible interpretations requiring confirmation in studies with physiological measurements. Similarly, the maintenance of IMTP performance may be compatible with reduced neuromuscular fatigue, but it does not establish preservation of central drive. The absence of direct neural, metabolic, hemodynamic, or muscle-oxygenation measures prevents differentiation among central and peripheral explanations. From an applied perspective, IPC may warrant further investigation as a strategy for athletes who must perform again within a short recovery window, but the present pilot findings should not be interpreted as mechanistic evidence.

Several limitations must be acknowledged. First, although the sample size was informed by a priori power analysis, the present study included only 16 participants and should therefore be interpreted as a pilot trial. This relatively small sample may limit statistical precision, increase uncertainty around effect estimates, and reduce the generalizability of the findings. Second, the absence of a sham-IPC condition means that placebo or expectancy effects cannot be fully excluded. Third, physiological biomarkers, such as lactate, blood markers of muscle damage, inflammatory responses, or muscle oxygenation indices, were not assessed; therefore, the proposed mechanisms remain speculative. Fourth, the IPC protocol used a fixed cuff pressure of 220 mmHg rather than an individualized AOP-based approach. Although this standardized pressure was selected based on prior IPC literature, the actual occlusive stimulus may have varied between participants. Finally, given the number of performance outcomes analyzed, the possibility of Type I error inflation due to multiple comparisons should be considered. Future studies should use larger samples, sham-controlled designs, individualized IPC pressure prescription, physiological measurements, and pre-specified primary outcomes.

## Conclusions

In this randomized controlled pilot trial, IPC applied prior to CT was associated with more favorable neuromuscular performance at 6 h post-intervention, particularly in selected sprint, jump, and isometric strength outcomes. From a practical perspective, this strategy may be relevant in settings where athletes are required to recover and perform again within a short time frame, such as high-frequency training schedules, tournament play, or multi-day competition environments. However, these findings are preliminary and should be interpreted cautiously because of the small sample size, absence of a sham-IPC condition, and lack of physiological measurements. Larger sham-controlled studies are needed to confirm whether IPC can reliably optimize the recovery–potentiation timeline following CT.

## Supplemental Information

10.7717/peerj.21649/supp-1 Supplemental Information 1 Original data

10.7717/peerj.21649/supp-2 Supplemental Information 2 CONSORT checklist

## Additional Information and Declarations

### Data Availability

The following information was supplied regarding data availability: The raw data is available in the Supplemental Files.

### Clinical Trial Registration

The following information was supplied regarding Clinical Trial registration: ChiCTR2500114472.
