# Supplementary material for: Ischemic preconditioning enhances 6-hour sprint, jump, and isometric strength responses to complex training in resistance-trained males: a randomized controlled pilot trial
Source: PeerJ. 2026 Aug 21;14:e21649. doi: 10.7717/peerj.21649 (PMC13505524; doi:10.7717/peerj.21649)
Supplement: Supplementary file 1 [file peerj-14-21649-s002.pdf]

CONSORT 2010 Checklist (Completed) – Supplemental File S2

Manuscript: Delayed potentiation effects of ischemic preconditioning combined with complex training on power and speed performance: a randomized controlled pilot trial

Note: Locations are provided by manuscript section/heading and figure/table number (page numbers may vary after journal typesetting).

Section/Topic	Item No.	Checklist item	Reported on (location in manuscript)
Title/Abstract	1a	Identification as a randomized trial in the title	Title indicates randomized controlled pilot trial
Title/Abstract	1b	Structured summary of trial design, methods, results, and conclusions	Abstract (Objective/Methods/Results/Conclusions)
Introduction	2a	Scientific background and explanation of rationale	Introduction
Introduction	2b	Specific objectives or hypotheses	Introduction (final paragraph)
Methods	3a	Trial design (e.g., parallel, allocation ratio)	Materials & Methods → Participants and study design (ADD: specify parallel design and allocation ratio 1:1 if not explicit)
Methods	3b	Important changes to methods after trial commencement	Not reported (assumed none; add statement if applicable)
Methods	4a	Eligibility criteria for participants	Materials & Methods → Participants and study design (inclusion/exclusion criteria)
Methods	4b	Settings and locations where data were collected	Materials & Methods → Measurements (testing setting; add location if missing)
Methods	5	Interventions for each group with sufficient details	Materials & Methods → Participants and study design + protocol details (add more if needed)
Methods	6a	Completely defined pre-specified primary and secondary outcome measures	Materials & Methods → Measurements (ADD: explicitly label primary vs secondary outcomes if not stated)
Methods	6b	Any changes to trial outcomes after commencement	Not reported (assumed none; add statement if applicable)

Methods	7a	How sample size was determined	Materials & Methods → Participants and study design (G*Power paragraph)
Methods	7b	When applicable, explanation of interim analyses and stopping guidelines	NA (pilot trial; no interim analysis)
Methods	8a	Method used to generate the random allocation sequence	Materials & Methods → Participants and study design (ADD: random sequence generation details)
Methods	8b	Type of randomisation; details of any restriction	Materials & Methods → Participants and study design (ADD: block/stratified or simple randomization)
Methods	9	Mechanism used to implement the random allocation sequence (allocation concealment)	Materials & Methods → Participants and study design (ADD: allocation concealment details)
Methods	10	Who generated the sequence, who enrolled participants, who assigned interventions	Materials & Methods → Participants and study design (ADD: roles)
Methods	11a	If done, who was blinded after assignment and how	Materials & Methods → Participants and study design (ADD: blinding statement)
Methods	11b	If relevant, description of similarity of interventions	NA/Not applicable (different conditions)
Methods	12a	Statistical methods used to compare groups for outcomes	Materials & Methods → Statistical Analysis
Methods	12b	Methods for additional analyses	Materials & Methods → Statistical Analysis (if subgroup/sensitivity, specify; otherwise NA)
Results	13a	Participant flow (numbers assigned, received, analyzed) for each group	Figure 1 (CONSORT flow diagram); Results first paragraph
Results	13b	Losses and exclusions after randomisation, with reasons	Figure 1 (all 0)

Results	14a	Dates defining recruitment and follow-up periods	Materials & Methods (ADD: recruitment/follow-up dates if not stated)
Results	14b	Why the trial ended or was stopped	NA (completed as planned)
Results	15	Baseline demographic and clinical characteristics for each group	Results + Table 1 (baseline characteristics; ensure table present)
Results	16	Number of participants included in each analysis and whether by original assigned groups	Results first paragraph (add ITT/PP statement if needed)
Results	17a	Results for each outcome with effect size and precision	Results (ANOVA outcomes)
Results	17b	For binary outcomes, absolute and relative effect sizes	NA
Results	18	Results of any other analyses	Results (if any; otherwise NA)
Harms	19	All important harms or unintended effects	ADD: include adverse events/harms statement (even if none)
Discussion	20	Trial limitations	Discussion (ensure included)
Discussion	21	Generalisability (external validity)	Discussion (ensure included)
Discussion	22	Interpretation consistent with results, balancing benefits and harms	Discussion (ensure included)
Other	23	Registration number and name of trial registry	Abstract (Methods) + Materials & Methods (Ethics/Design) (add ChiCTR2500114472)
Other	24	Where the full trial protocol can be accessed, if available	ADD: State “Protocol available as Supplemental File S1” in Methods or Data Availability
Other	25	Sources of funding and role of funders	Funding/Declarations section (ensure included)
